# Hypoxia-Induced miR-210 Overexpression Promotes the Differentiation of Human-Induced Pluripotent Stem Cells to Hepatocyte-Like Cells on Random Nanofiber Poly-L-Lactic Acid/Poly (*ε*-Caprolactone) Scaffolds

**DOI:** 10.1155/2021/4229721

**Published:** 2021-11-22

**Authors:** Naser Mobarra, Sara Raji, Sara Najafi, Farzaneh Kamelan Kafi, Gordon A. Ferns, Reza Pakzad

**Affiliations:** ^1^Department of Laboratory Sciences, School of Paramedical Sciences, Mashhad University of Medical Sciences, Mashhad, Iran; ^2^Persian Cohort Research Center, Mashhad University of Medical Sciences, Mashhad, Iran; ^3^Student Research Committee, Faculty of Medicine, Mashhad University of Medical Sciences, Mashhad, Iran; ^4^Brighton and Sussex Medical School, Division of Medical Education, Brighton, UK; ^5^Department of Epidemiology, Faculty of Health, Ilam University of Medical Sciences, Ilam, Iran

## Abstract

An alternative treatment to liver transplantation includes the use of differentiated stem cells. Hypoxia has been shown to endow human-induced pluripotent stem cells (hiPSCs) with enhanced hepatic differentiation. We have investigated a new strategy for hepatocyte differentiation from hiPSCs using a three-step differentiation protocol with lentiviral overexpression of hypoxia-microRNA-210 of cells grown on a hybrid scaffold. We analyzed the transduction of the miR-210 lentiviral and definitive endoderm and pluripotency gene markers, including SRY-box 17 (SOX17), forkhead box A2 (FOXA2), and octamer-binding transcription factor 4 (OCT-4) by Real-Time PCR and fluorescent microscope. The scanning electron microscopy (SEM) examined the 3D cell morphological changes. Immunocytochemistry staining was used together with assays for aspartate aminotransferase, alanine aminotransferase, and urea secretion to analyze hepatocyte biomarkers and functional markers consisting of *α*-fetoprotein (AFP), low-density lipoprotein (LDL) uptake, fat accumulation, and glycogen. The flow cytometry analyzed the generation of reactive oxygen species (ROS). Compared to cells transfected with the blank lentiviral vectors as a control, overexpressing miR-210 was at higher levels in hiPSCs. The expression of endodermal genes and glycogen synthesis significantly increased in the differentiated lentiviral miR-210 cells without any differences regarding lipid storage level. Additionally, cells containing miR-210 showed a greater expression of ALB, LDL, AST, ALT, urea, and insignificant lower AFP and ROS levels after 18 days. However, SEM showed no significant differences between cells under the differentiation process and controls. In conclusion, the differentiation of hiPSCs to hepatocyte-like cells under hypoxia miR-210 may be a suitable method for cell therapy and regenerative medicine.

## 1. Introduction

Liver transplantation is the only currently available treatment for end-stage liver disease. Approximately a million people suffer from liver failure and die annually. However, there are difficulties associated with liver transplantation, including lack of liver donors, long waiting times, and high costs [1, 2]. Hence, further strategies are required to deal with this condition. The transplantation of tissue-derived stem cells is one possible solution to mitigate liver disease, and there has been substantial research on this topic [3]. In regenerative medicine, human-induced pluripotent stem cells (hiPSCs) are sources of stem cells utilized in artificial cell engineering [1, 4]. HiPSCs resemble embryonic stem (ES) cells but can be produced from mature somatic fibroblast cells. Research has explored the ability of these pluripotent cells to differentiate into various types of tissue cells in vitro conditioned with an extracellular matrix [4, 5]. HiPSCs were considered multipotent with the potential to be aimed toward differentiation into human hepatocytes [6]. Since the environment of stem cells plays an essential role in their function due to cell-matrix contacts, the culture of hiPSCs on nanofibrous scaffolds has a prominent potential to proliferate and differentiate into hepatocytes. Nano-scale poly-L-lactic acid/poly (*ε*-caprolactone) (PLLA/PCL) fibers are one of the available three-dimensional (3D) culture mediums. These biocompatible polymers can provide a pliable and wide surface suitable for cell attachment. Also, it does not activate the immune response [7]. In regenerative medicine, PLLA/PCL can provide a stable, bioresorbable, flexible, and safe material for the differentiation of hiPSCs [8, 9]. Several studies have examined the efficacy of different methodologies for the differentiation of hepatocytes [10–13]. Particular genes and microRNAs such asmiR-375 and miR-122 are supposed to be accountable for hepatocyte differentiation [14]. MicroRNAs (miRNAs) are noncoding RNA species, acting as gene expression modulators by affecting their target mRNAs' translation potency or steadiness. Recent breakthroughs regarding miRNAs have highlighted their significant impact on cellular proliferation and differentiation [15, 16]. Among these, microRNA-210 (miR-210) has attracted particular attention due to its upregulation and antiapoptotic action during hypoxia, categorizing it as a hypoxamiR [17, 18]. According to evidence, the miR-210 expression level increased during the establishment of epithelial polarity in the course of differentiation [19].

Moreover, according to a recent study, miR-210 is upregulated under oxidative stress and has antiapoptotic and prosurvival functions. Reducing reactive oxygen species (ROS) production and downregulating the (caspase-8-associated protein 2) CASP8AP2 pathway are some of the miR-210 mechanisms detected against oxidative stress [20]. Formerly, hypoxia was found to increase the expression of cellular markers and improve its function [21]; however, there were no investigations on hepatocytes. Through in vitro experiments, cells and tissue were commonly cultured at atmospheric oxygen concentration, though differentiation of stem cells was performed at a lower oxygen level in vivo. Consequently, we primarily endeavored to provide the most analogous condition with the human body to expose cells under hypoxia via miR-210 [22]. To the best of our knowledge, no one has investigated the effect of miR-210 inducing hypoxia on hepatocyte differentiation and oxidative stress changes during this process. This present study was an extension to examine whether lentiviral overexpression of miR-210 mediated on 3D PLLA/PCL nano-scaffold can induce hepatocyte differentiation from hiPSCs. Research has also evaluated the impact on oxidative stress changes.

## 2. Materials and Methods

### 2.1. Lentiviral miRNA Production

#### 2.1.1. HEK-293T Cell Line Culture

Approximately, 4 × 10^6^ HEK-293T (T-293) cells were cultured in a 100 mm Petri dish for 24 hours before transfection with viral constructs at the beginning of packaging. The cells were cultured in Dulbecco's modified Eagle's medium (DMEM, Gibco, 31330038, USA), 10% fetal bovine serum (FBS, Gibco, 10270106, USA), 2 mM L-glutamine (Gibco, 25030, USA), 100 U/mL penicillin, and 100 *μ*L streptomycin (Gibco, 15140–122, USA). Cells were maintained at 37°C in the CO_2_ incubator until 70% confluent. Replacement of the culture medium with fresh medium took place two hours before transfection.

#### 2.1.2. Plasmid Mixture for Packaging

21 *μ*g of pLEX–TurboGFP-miR210 vector (495 *μ*L), 21 *μ*g of psPAX2 plasmid (112 *μ*L), and 5.10 *μ*g of pMD2.G (14 *μ*L) plasmid (all from Stem Cell Technology Research Center, Tehran, Iran) were mixed in a 50 mL Falcon tube. The addition of sterile deionized water is aimed at reaching a total volume of 912 *μ*L.

#### 2.1.3. Preparation of Reactive Calcium Phosphate Buffer

DNA plasmids were combined with 105 *μ*L of 2.5 M calcium chloride and 33 *μ*L TE buffer (1x) in sterile deionized water (912 *μ*L), in a 50 mL Falcon tube to produce virus from a 100 mm Petri dish containing HEK T-293 cells. The Falcon tube was mixed with vortex, along with dropwise addition of 1050 *μ*L of 2x HBSS buffer during vortex mixing. The mixture was incubated at room temperature for 10-15 minutes until it became opaque, followed by its dropwise addition to the entire surface of the HEK T-293 cells plate. Subsequently, the cells were incubated for 16 hours with 5% CO^2^ at 37°C. The pLenti-III-GFP-negative control cells also underwent this process.

#### 2.1.4. Virus Production and Harvest

The medium was replaced with fresh Dulbecco's modified Eagle's medium (DMEM) containing 10% FBS after 16 h incubation, followed by harvesting the cell medium containing viruses every 24 hours. This process lasted for 72 hours, after which the harvested viruses were concentrated at 1800 rpm for 10 minutes and filtered under 0.4 mm pore size to separate cell debris. Finally, the culture containing viruses was divided into small aliquots on ice and transferred to a -70°C freezer.

#### 2.1.5. HiPSC Cell Culture

As previously reported [10], human-induced pluripotent stem cells were cultivated and expanded on mouse embryonic fibroblasts (MEFs) as feeders, inactivated with mitomycin-C. These cells were enriched with 10% knockout serum replacement, 100 *μ*M nonessential amino acids, 2 mM l-glutamine, 100 *μ*M 2-mercaptoethanol, and 4 ng/mL basic fibroblast growth factor (bFGF, totally from Invitrogen) in DMEM/nutrient mixture F-12. HiPSC colonies were detached with 1 mg/mL collagenase IV (Gibco, 15140-122), incubated for 5-15 min, and then isolated slowly from MEFs to form an embryonic body [10].

#### 2.1.6. Embryonic Body Formation and Lentiviral MicroRNA Transduction

The hiPSC colonies were divided into three parts. In part A, 10 *μ*L of the concentrated GFP positive virus-containing miR-210 target gene and 17 *μ*L of the EB medium were transduced into 105 hiPSCs cells based on the multiplicity of infection (MOI) of 20. Part B was identical to part two, except using a GFP-positive virus without the miR-210 gene as an empty vector (Neg). Finally, in part C, 10^5^ cells were placed in 27 *μ*L of the EB medium as a control group. The EB formation took place using the hanging drop method [23]. Of note, the EB medium contained the same nutrients as the hiPSC medium without bFGF, 2ME, and antibiotics. After two days, EBs were transformed into less adhesive 100 mm plates for additional three days after washing with PBS while exchanging the medium if needed. Positive, clear, and large EBs were transformed into PLLA/PCL nanofiber cellular culture 4-well plates after quantitative and qualitative confirmation of the virus in the EBs to differentiate into endoderm and hepatocyte-like cells with the help of growth factors. The final step included the addition of 2 *μ*g/mL puromycin for 8 hours to select cells with the virus.

#### 2.1.7. Quantitative and Qualitative Assessment for Lentiviral Production

Real-time PCR was used to evaluate the miR-210 expression in the transduced EB cells compared to cells containing the empty vector virus. Total RNA was extracted using Trizol Reagent (Gibco, 15596018), measuring RNA concentration by nano-drops. miRNA 1st-Strand cDNA synthesis (Fermentas k1621) was performed 1 *μ*g of total RNA and specific stem-loop primer (supplementary Figure S1 and Table S1). The qRT-PCR was carried out using SYBR Premix ExTaq Master (Takara, RR820A), at 95°C for 2 min, along with 40 cycles of 95°C for 5 seconds and extension at 60°C for 45 seconds. Snord 47 was used as an internally originating control gene for normalization (Table 1). Data were analyzed using the comparative CT method expressed relative to EB cells containing the empty vector. The lentiviral GFP fluorescent protein assay in EB cells was done for the qualitative assay before differentiation started.

### 2.2. Tissue Engineering

#### 2.2.1. Hybrid PLLA/PCL Nanofibrous Preparation, Surface Treatment, and Sterilization

Random scaffolds were manufactured by electrospinning in the Stem Cell Technology Center (Tehran, Iran) [1]. PLLA (0.4 g, MW: 18 kDa) and PCL (1.22 g, MW: 14 kDa) powder, dissolved in N, N-dimethylformamide solvents plus chloroform, were homogenized using a needle. There was a collector at a distance of 15 cm from the needle. The needle and the collector received a high voltage potential (20 kV). Disposable syringes containing a polymer solution were forced through the needle and collected as nanofibrous on the rotating cylinder. Afterward, a plasma generator with a low 40 kHz frequency was applied for oxygen treatment in a cylindrical quartz reactor to enhance the scaffold's hydrophilic characteristics (Diener, Electronics, Ebhausen, Germany). Pure oxygen was introduced into the reaction chamber at 0.4 mbar pressure, and then, the glow discharge was performed for 3 min. Plasma-treated nanofibrous sheets were punched to make rims with a 0.5 cm diameter. Ethanol (70%) was used to sterilize the manufactured circular scaffold for 12 hours, which was also rinsed with PBS three times and left overnight in the complete culture medium.

#### 2.2.2. Endoderm, Premature, and Maturation Steps of HLC Differentiation

Differentiation into endoderm cells was the first stage of hepatic differentiation from hiPSC cells. All three groups of EB cells produced were placed in 4-well adhesive plates containing 3D nanofibrous PLLA/PCL scaffold for 2 hours to get sufficient time for adhesion. After that, based on a previous report [1], 500 *μ*L of complete endoderm culture medium containing 5 mL RPMI-1640, 10 ng/mL Activin A, and 5 *μ*L B27 (1x) was added. Cell culture lasted for three days while exchanging the medium every day. Then foxa2, sox17, and OCT-4 genes were evaluated using the q-PCR at this stage. In the second stage, the culture medium was replaced by a new medium containing 10 mL hepatocyte culture medium (HCP), 20 ng/mL hepatocyte growth factor (HGF), 20 ng/mL fibroblast growth factor (FGF-4), and 2% FBS for premature formation. Finally, the medium was altered and lasted longer (9 days) during the maturation and production of hepatocyte-like cells. At the end of the final step, the cells were evaluated in terms of morphology, the production of biochemical markers, and molecular and immunocytochemistry (ICC) staining.

#### 2.2.3. RNA Extraction and qPCR for Endodermal Genes

Trizol reagent (Gibco, 15596018) was used to extract the total RNA on days 0 and 3 in cells differentiated with lentiviral miRNA-210 and empty vector to endoderm differentiation onset. Based on the manufacturing protocol, 2 *μ*g of total RNA was utilized for the cDNA synthesis (Fermentas, k1621). The qRT-PCR was carried out using TaKaRa SYBR Premix ExTaq Master (Takara, RR820A) at 95°C for 2 min, using 40 cycles of 95°C for 5 s and extension at 60°C for 45 s. Beta actin was used as an internally originating control gene and for normalization. Data were analyzed using the comparative CT method, expressed relative to undifferentiated hiPSCs (day 0). Stem Cells Technology Research Center, Tehran, Iran, provided the applied endodermal primers presented in Table 2.

### 2.3. Flow Cytometry

#### 2.3.1. Recombinant Retrovirus Titer Determination

Approximately 6 × 10^4^ HEK-293T cells were plated into 4-wells of 24-well plates to determine titration of produced viruses. The serial amounts of 1, 4, and 16 *μ*L viruses were added, respectively, and the fourth well was considered as the control group. The plate was shivered slowly and incubated at 37°C in 5% CO^2^ for four days. Flow cytometry assay was used to evaluate virus titration 24, 48, and 72 hours after transduction in addition on day 5 of EB formation in three phases: empty EB, empty vector (control), and EB containing lentiviral miR-210.

#### 2.3.2. ROS Detection

Intracellular ROS and oxidative stress changes during differentiation were detected using 2′, 7′-dichlorofluorescein-diacetate as a fluorescent probe converted into the nonfluorescent product by cellular esterase. It was subsequently converted into 2′, 7′–dichlorofluorescein (DCF) after being oxidized by intracellular ROS, resulting in a highly fluorescent dye that was detectable via flow cytometer. Examination of 1.5 × 10^4^ cells was the first step. The cells were rinsed twice with PBS and trypsinized, respectively, after removal of the cell culture medium. Besides, they were centrifuged at 1200 rpm for 5 minutes, followed by their resuspension in 0.5 mL of diluted buffer with 10% FBS before incubation at 37°C for three hours. The addition of 5 *μ*L of DCFDA was ahead of 3 hours incubation once more. The device calculated the amount of ROS production, and the FlowJo software was used to illustrate the graph.

#### 2.3.3. Biochemical Assays for AFP, AST, ALT, and Urea Production

A total of 10^5^ mature supernatant cells were assessed using a chemiluminescence immunoassay method kit (Diasorin, Saluggia, Italy) for assay AFP production. Moreover, AST, ALT, and urea levels of the cell supernatants were assessed by an auto-analyzer and colorimetric methods on days 9 and 18 using a Colorimetric Assay Kit (1400029; Pars Azmun) based on the manufacturer's instructions with a sensitivity of 2 IU/L (for ALT, AST) and 2 mg/dL (for urea). Undifferentiated hiPCSc media made up the controls.

### 2.4. Immunocytochemistry Staining

#### 2.4.1. Alpha-Fetoprotein and Albumin

Immunocytochemistry evaluated the specific hepatic markers after the induction of differentiation. According to the protocol, the HLC-derived cells were rinsed with PBS and fixed with 4% paraformaldehyde (Sigma-Aldrich, 30525-89-4) for 15 min at 4°C, followed by 5 min at room temperature. After the fixative removal, cells were washed with PBS three times, and Triton X-100 was used to permeabilize cells in PBS. They were incubated with primary mouse monoclonal antibodies with a 1 : 200 dilution against *α*-fetoprotein (MAB1368; R&D) and albumin (MAB1455; R&D) overnight at 4°C. Next, the cells were rinsed with PBS-Tween 20 (0.1%) three times. They were also incubated with a secondary antibody, anti-goat mouse (1 : 100; F0102B; R&D), and phycoerythrin-conjugated [24] properly in the darkroom at 37°C for 60 min. Then, these matured cells were rinsed with PBS, and the nuclei eventually were counterstained with 4′, 6-diamidino-2-phenylindole ((DAPI) (1 *μ*g/mL)) (Sigma-Aldrich, 28718-90-3) for 1 min. A fluorescence microscope (Nikon; Japan) was utilized to take images.

#### 2.4.2. LDL Uptake

A DiI-Ac-LDL staining kit was used according to the manufacturer's guidance (Biomedical Technologies, BT-904) to assess the low-density lipoprotein (LDL) uptake. First, 10 *μ*g/mL of DiI-Ac-LDL was dissolved in DMEM, foiled, and incubated at 37°C for 4 hours, followed by its washing with PBS three times and exposure to DAPI for about 15-30 seconds. Finally, they were rinsed and evaluated by a fluorescent microscope (Nikon; Japan).

#### 2.4.3. Periodic Acid-Schiff (PAS) Stain for Glycogen Synthesis

In the first step, the differentiated and fixed cells were exposed to 1% periodic acid (Sigma, 10450-60-9) for 5 min and washed 3x with PBS. Afterward, they were treated with Schiff's reagent (Sigma, 3952016) for 10-15 minutes and rinsed in running water. Finally, the cells were assessed by light microscopy using 100x magnification (Olympus, Japan).

#### 2.4.4. Oil Red

Differentiated cells were fixed with 4% paraformaldehyde for 45 min at room temperature to assess lipid storage levels and observe lipid granules. The cells were subsequently rinsed with PBS and exposed to oil red reagent for 45 min at room temperature. Then, those cells were washed with running water and analyzed by a light microscope using 100x magnification (Olympus, Japan).

#### 2.4.5. Scanning Electron Microscopy (SEM)

HiPSCs and derived HLCs cells were washed three times with PBS and fixed in 2.5% vol/vol glutaraldehyde for an hour, followed by their dehydration in a series of graded ethanol (10-100%). The fixed cells were dehumidified, exposed to air at room temperature overnight, and sputter-coated with gold using a sputter coating unit (MED010; Baltec, Pittsfield, MA) before observation under the scanning electron microscopy (SEM, MED010; Bal-tec, Pittsfield, MA) at different magnifications. A Hitachi Model-4500 SEM (Hitachi, Chiyoda, and Tokyo, Japan) with an acceleration of 20 kV voltage was used to view the images.

#### 2.4.6. Statistical Analyses

The Kolmogorov–Smirnov test evaluated the distribution of values. Normal quantitative and qualitative data are expressed by mean ± SD and mean ± SEM, respectively. The log fold change showed alterations of hepatocyte-specific gene markers. Biochemical and oxidative stress analyses were conducted by ANOVA and Student's *t*-test to compare all groups. Tukey post hoc was also performed for pairwise comparison between two groups, in case of being significant. A *P* value of < 0.05 showed significance. The statistical procedures were conducted by SPSS 16 (SPSS Inc., Chicago, IL, USA).

## 3. Results

### 3.1. Production and Evaluation of the Viral Load

**Figure 1:**
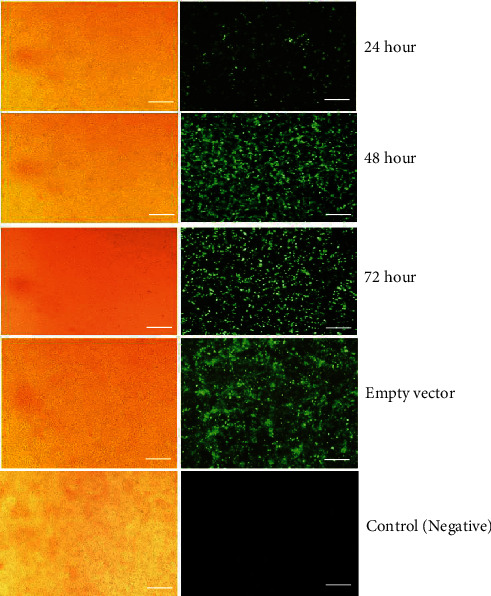
Light microscope (a) and green fluorescent protein (GFP) (b) expression in 293T-cells infected with miRNA-210 lentiviral vectors in the test group 24, 48, and 72 hours after transduction, without lentiviral vectors (empty vector after 72 hours) and control group (negative). Scale bars are 100 *μ*m.

**Figure 2:**
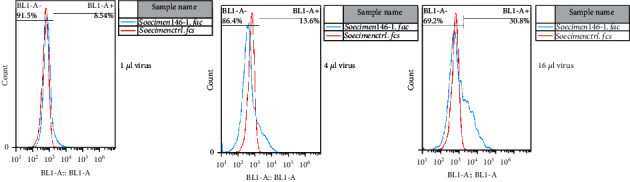
Percentages of viral transduction with concentrations of 1, 4, and 16 microliters, leading to the values of 8.5%, 13.6%, and 30.8%, respectively.

The miR-210 lentiviral vectors and the blank lentiviral vectors were packed into 293T-cell to produce retrovirus expressing miR-210. The supernatant of the cell culture, which contained the retrovirus, was collected at 24, 48, and 72 hours.  Figure 1 shows the results of the fluorescent microscope examination of virus-producing 293T-cell at those times. The qualitative results demonstrated that the virus's expression increased remarkably every 24 hours after transfection until 72 hours. The retroviruses were collected, and the viral titer was measured by adding 1, 4, and 16 microliters of the virus to 5 × 10^4^ different 293T-cells based on the Murano protocol. After four days, the concentration of the infected cells was measured by flow cytometry. The viral titer results in 1, 4, and 16 microliters were 8.5%, 13.6%, and 30.8%, respectively. The best viral titer for transduction to hiPSCs was 1.925108 TU/mL.  Figure 2 shows the results of the viral titration flow cytometry.

### 3.2. HiPSC Culture and EB Formation

**Figure 3:**
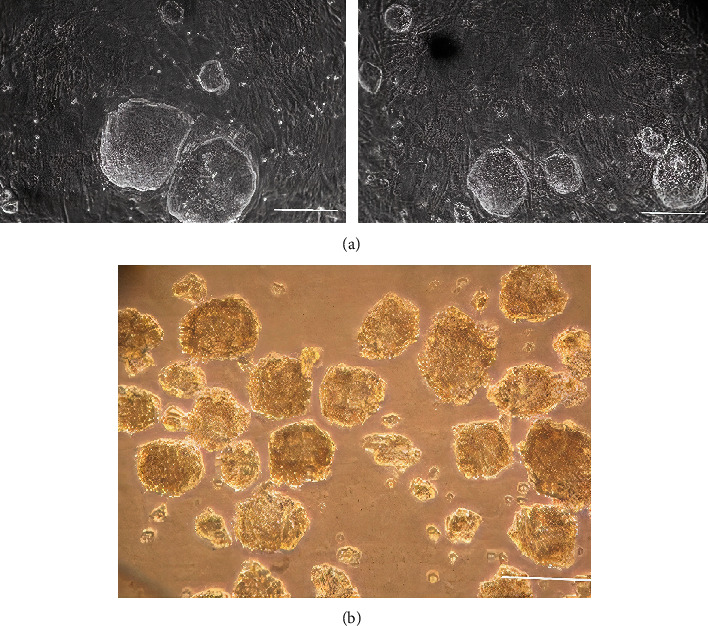
Cultured human-induced pluripotent stem cells (hiPSCs) on mouse embryonic fibroblasts (MEF) feeder cells (a) and fluorescent microscopy of embryonic bodies (EB) formation (b). Scale bars are 100 *μ*m.

HiPSCs with smooth margins and flat colonies were grown on MEF feeder layers (Figure 3(a)). The colonies were separated and transferred under hanging drop optimal conditions to form embryonic bodies (EBs) (Figure 3(b)).

### 3.3. Lentiviral miR-210 Expression and Transduction Efficacy

**Figure 4:**
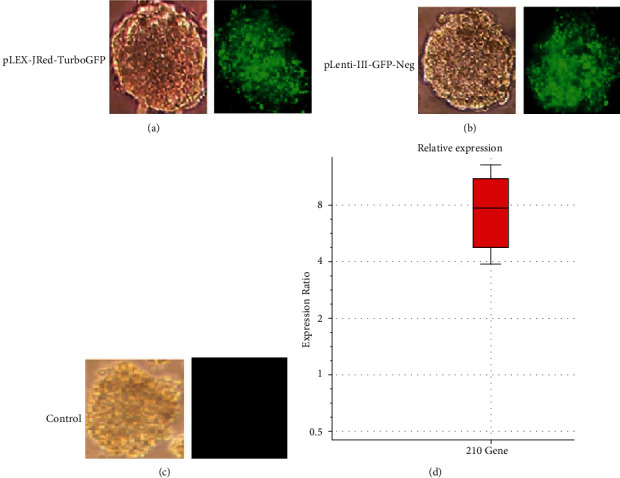
Phase contrast and fluorescence images of embryonic bodies (EBs) transduced with miR-210 lentiviruses (a), with blank lentiviruses (negative control) (b), and (c) the untransduced EBs (control). qPCR analysis of miRNA-210 expression in embryonic bodies after 48 hours of transduction (d).

Lentiviral transduction into colony hiPSCs was applied after determining the viral load for EB formation. HiPSCs were passaged, counted, and divided into three groups, two of which received viruses based on MOI = 20. The first group contained the miR-210 gene, while the second group received blank lentiviral vectors. The third group was free from any virus (Figures 4(a)–4(c)). Also, the expression of miR-210 was determined by Real-Time PCR after lentivirus transduction. Expression of miR-210 was approximately 7.11 times more in hiPSCs-miR-210 compared to the negative control group, demonstrating a significant difference (*P* < 0.001) (Figure 4).

### 3.4. Evaluation of Scaffold Characterization and Morphological Changes of hiPSCs to HLCs under Scanning Electron Microscopy (SEM)

**Figure 5:**
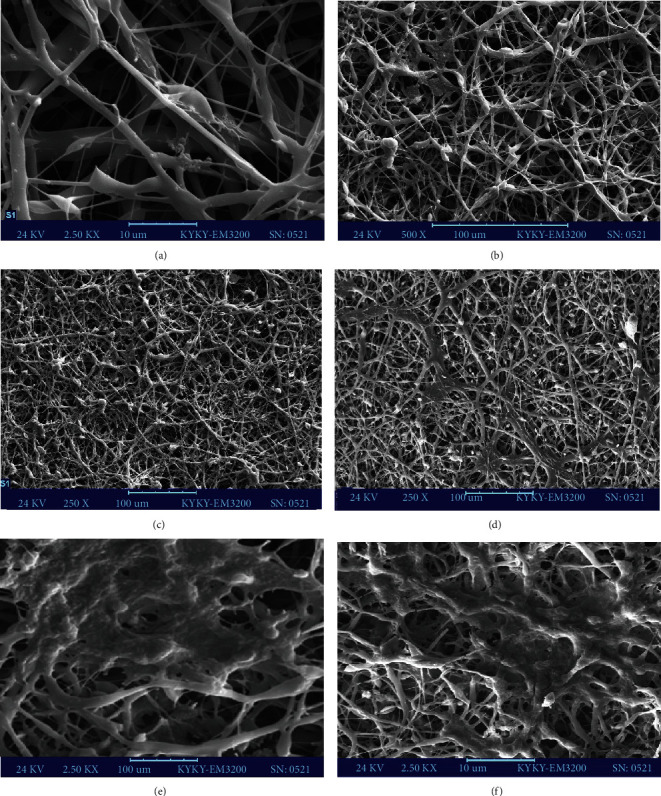
Poly-L-lactic acid-poly *ε*-caprolactone (PLLA/PCL) nanofibrous scaffold morphology under three different magnifications using scanning electron microscopy (SEM). The scaffolds had a perfectly porous structure with no defects, and the shape of the fibers was so flat, being applicable for tissue engineering (a–c). Morphological changes of 3D-miR (e) and 3D-non miR cells (f) 18 days after differentiation compared to iPS cells as a negative control group (d). There were no significant differences between iPS cells and virus-affected cells (3D-miR: group cultured on the three-dimensional scaffold with miR-210 transduction; 3D-non miR: group culture on the three-dimensional scaffold without miR-210 transduction). Scale bar = 10 *μ*m (a, e, f) and 100 *μ*m (b–d).

The scaffold and flat nanofibers' highly porous and nonwoven structures were viewed under the SEM, making this culture medium an appropriate choice for tissue engineering. The scaffold's pores and pits' diameters were adequate to increase surface area to volume ratio and cell adhesion proportionally. The mean size of the fibers was 90-95 nm (Figures 5(a)–5(c)). On day 18, observations showed no remarkable differences between hiPSCs and virus-affected cells, except the cells containing pLEX miR-210 that were slightly out of flattened shapes and composed of round processes, unlike the pLEX-Neg group (Figures 5(d)–5(f)).

### 3.5. Evaluation of Endodermal Gene Marker Expression by Real-Time PCR

**Figure 6:**
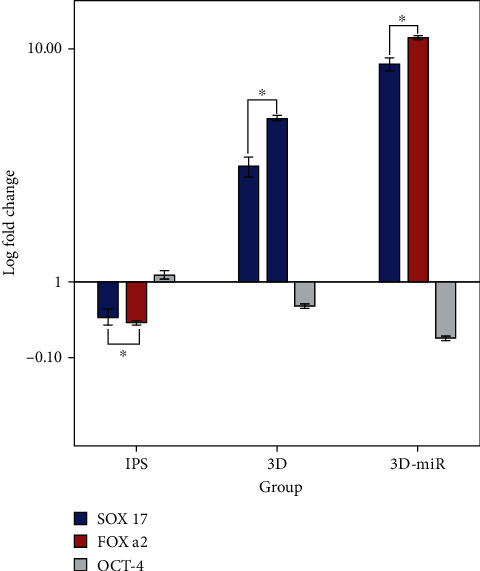
Real-time PCR analysis of relative endodermal gene marker expression (SOX17: SRY-box 17; FOXA2: forkhead box A2) and pluripotency marker (OCT-4: octamer-binding transcription factor 4). IPS was used to normalize the data. 3D-miR: group culture on the three-dimensional scaffold with miR-210 transduction; 3D: group culture on the three-dimensional scaffold without miR-210 transduction. ^∗^*P* < 0.001.

Supplementary Table S1 and Figure 6 present the mean of fold change (log) in different groups, including hiPS, 3D, and 3D-miR. According to this result, in the hiPSC cells, the means of SOX17, FOX a2, and OCT-4 were −0.23 ± 0.03, −0.29 ± 0.01, and 0.03 ± 0.01, respectively, indicating significant differences (*P* < 0.001). Similarly, there were remarkable differences in the mean between 3D and 3D-miR groups (*P* < 0.001). Upon Tukey's results, as shown in supplementary Table S1, the mean of SOX17 was higher than FOX a2 (*P* < 0.031) and lower than OCT-4 (*P* < 0.001) in the hiPS group. Our results have not reached statistically significant differences between SOX17 and FOX a2 in these three groups; however, the mean expression of SOX17 and FOX a2 in 3D-miR was significantly higher than in the 3D cells. Supplementary Table S1 and Figure 6 summarize other twofold comparisons.

### 3.6. Evaluation of Hepatocyte-Specific Marker Expression by Immunocytochemistry Staining

**Figure 7:**
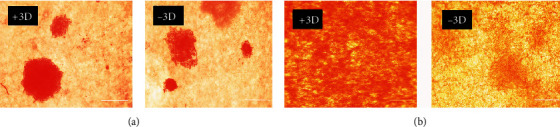
Lipid (a) and glycogen storage (b) analysis with oil red and PAS staining on the nanofibrous scaffold with lentiviral miR-210 transduction (+3D) and without miR-210 (-3D). Data are shown as mean ± SEM. Scale bar = 100 *μ*m. PAS: periodic acid-Schiff; miR-210: microRNA-210.

**Figure 8:**
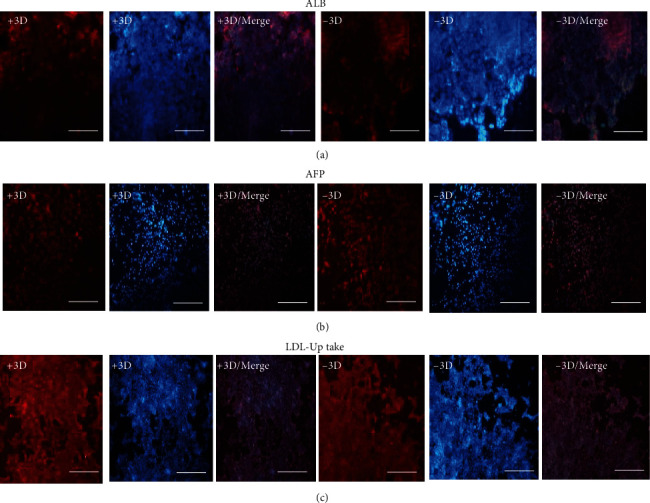
Immunocytochemistry staining (a) ALB and (b) AFP protein, as well as (c) LDL-uptake, the main hepatic markers in end-stage–derived cells on the nanofibrous scaffold with lentiviral miR-210 transduction (+3D) and without miR-210 (-3D). Nuclei were stained with 4′,6-diamidino-2-phenylindole (DAPI). Data are presented as mean ± SEM. Scale bar = 100 *μ*m. AFP: *α*-fetoprotein; ALB: albumin; LDL: low-density lipoprotein.

Hepatocyte-specific markers, including lipid concentration and glycogen storage, albumin (ALB), alpha-fetoprotein (AFP) expression, and LDL uptake ratio, were evaluated on the 18th day of differentiation by immunocytochemistry staining. Oil red analysis did not show any differences regarding lipid content among 3D-miR and 3D-non miR groups (Figure 7(a)). On the other hand, glycogen staining (PAS) analysis on the 18th day of differentiation inducement showed a significant difference between 3D-miR210 compared to 3D-non miR cells (Figure 7(b)). hiPSCs transduced derived-hepatocytes with miR-210 (+3D) expressed ALB, and LDL-uptake ratio markers were greater than the hiPSCs without recombinant virus (-3D) (Figure 8). Of note, HLCs in the 3D-miR group expressed slightly lower levels of AFP than the 3D-non miR group on day 18, which was not remarkable (Figure 8(b)). Moreover, culturing 3D nano-scaffolds with miR-210 increased the potency for better differentiation compared to 3D-non miR. These findings indicated that PLLA/PCL scaffold with miR-210 could enhance and improve the hiPSC differentiation efficiency to HLCs.

### 3.7. Biochemical Analyses

**Figure 9:**
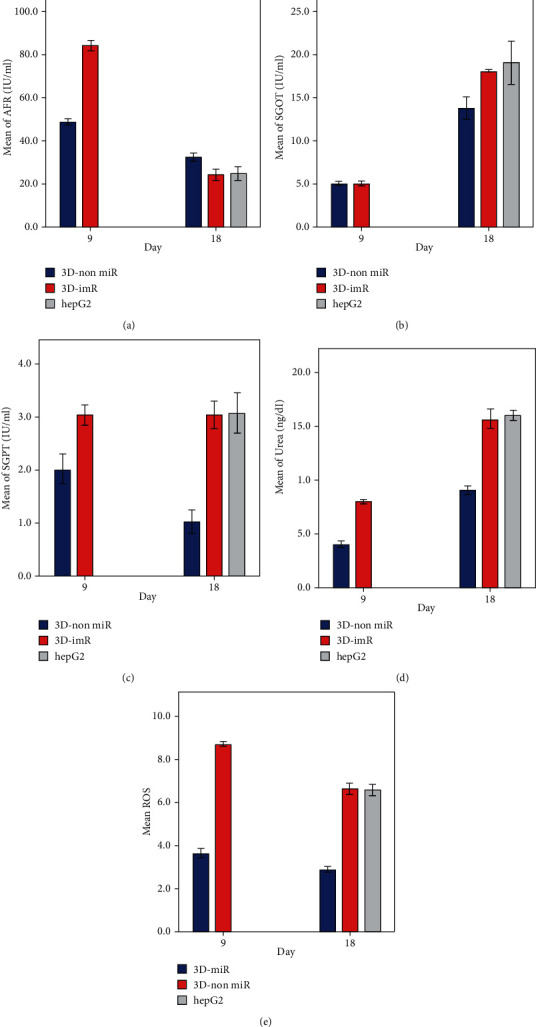
AFP (a), SGOT (b), SGPT (c), urea concentration (d), and mean of ROS (e) in 3D-miR and 3D-non miR groups on the 9th and 18th days of differentiation, compared to hepG2 cells as the positive control group on day 18. Data are shown as means ± SD. On day 18, the 3D-miR group showed a significant increase compared to the 3D-non miR group regarding SGPT, SGOT, and urea concentration; however, AFP and ROS levels were higher in 3D-non miR cells. 3D-miR: group culture on the three-dimensional scaffold with miR-210 transduction; 3D-non miR: group culture on the three-dimensional scaffold without miR-210 transduction. ^∗^*P* < 0.001. AFP: *α*-fetoprotein; SGPT: serum glutamic pyruvic transaminase; SGOT: serum glutamic-oxaloacetic transaminase.

Urea, SGPT (serum glutamic pyruvic transaminase), or alanine aminotransferase and SGOT (serum glutamic-oxaloacetic transaminase) or aspartate transaminase secretion and AFP concentration were assessed by the auto-analyzer and chemiluminescence immunoassay test, respectively, in 3D-miR and 3D-non miR nanofiber matrix cells after 9 and 18 days of differentiation. Additionally, these biochemical factors were compared with HepG2 cells as a positive control group on the 18th day. Supplementary Table S2 and Figure 9(a) show AFP's mean on days 9 and 18 in two 3D-miR and 3D-non miR groups. The maximum AFP expression was in the 3D-miR group on day 9, which significantly differed from the 3D-non miR group (*P* < 0.001; 84.12 ± 1.02 IU/mL vs. 48.81 ± 0.61 IU/mL). Moreover, there was an evident difference between the three groups on day 18 (*P* < 0.001). The expression of AFP increased by mean ± SD in 3D-non miR compared to 3D-miR and hepG2 groups (*P* < 0.001, *P* < 0.001), but there were no significant differences between 3D-miR and hepG2 cells (*P* = 0.649, 24.12 ± 1.02 vs. 24.83 ± 1.26) (Supplementary Table S2). AST activity significantly increased on day 18 compared to day 9. There was a significant difference between the hepG2 and 3D-non miR groups on day 18 (*P* < 0.001, 13.76 ± 0.52 vs. 18.97 ± 1.01). However, there were no differences between 3D-miR and 3D-non miR groups on day 9 (*P* = 0.998, 5.03 ± 0.11 vs. 5.03 ± 010). (Supplementary Table S2, Figure 9(b)). In the measurement of ALT release (Supplementary Table S2, Figure 9(c)), there was a significant difference between groups on days 9 and 18 (*P* < 0.001, *P* < 0.001). Nevertheless, ALT activity in the 3D-miR group was similar on both days (3.03 ± 0.08 vs. 3.03 ± 0.1). The secretion level of ALT was lower in 3D-non miR compared to other groups on day 18.

Evaluation of the urea production demonstrated a significant difference between cells on both days (*P* < 0.001, *P* < 0.001). The maximum production was in the hepG2 group on day 18. Also, there was a significant reduction of urea in 3D-non miR compared to 3D-miR and hepG2 groups (*P* < 0.001, *P* < 0.001) (Supplementary Table S2, Figure 9(d)).

### 3.8. Flow Cytometry Analysis

Oxidative stress status was investigated by flow cytometry analysis. As shown in Figure 9(e) and Supplementary Table S3 on day 9, ROS mean was significantly different between 3D-non miR and 3D-miR groups (*P* < 0.001). Furthermore, there was a significant difference between 3D-non miR, 3D-miR, and hepG2 groups on day 18 (*P* < 0.001). Levels of ROS in the 3D-miR group were lower compared to 3D-non miR (*P* < 0.001) and hepG2 groups (*P* < 0.001) on day 18. The mean of ROS was not significantly different between the 3D-miR and hepG2 groups (*P* = 0.820).

## 4. Discussion

We investigated a new pathway for hiPSC differentiation into hepatocytes on 3D scaffolds using miR-210, upregulated in response to hypoxia in various types of cells [25, 26]. Preliminary studies elucidated more beneficial outcomes of 3D culture compared to routine culture in cell differentiation [27]. Other investigations also indicated that using nanofibrous scaffolds as a 3D environment may resemble natural ECM and enhance stem cell interactions with surrounding cells or materials, leading to preferable differentiations [28]. Experiments on several 3D components exhibited that hybrid scaffolds such as PLLA/PCL hybrid scaffold function better than single ones to stabilize cells, maintain their flexibility, and support hiPSCs proliferation [29]. According to the established effects and our previous studies, the nanofibrous PLLA/PCL hybrid scaffold was appropriately used as a matrix for differentiation of hiPSCs to HLCs in the current study.

Recently, there has been much attention to HiPSCs driven from mature fibroblasts for use in liver tissue repair and as an alternative solution for the problems of whole organ transplantation [30]. A strategy for enhancing differentiation and producing the most similar cells to hepatocytes is one of the main issues of this field. There is a great need for a culture medium with the oxygen level of the body, which can be provided by microRNAs especially miR-210. Recently, miRNAs have been recognized to contribute to different processes, including mRNA translation, stem cell renewing, replication, and especially hepatocyte differentiation [14, 31]. Significant elevation of miR-210 expression under physio-pathological statements such as hypoxia has made it interesting for research [18]. In this study we first cultured hiPSCs on MEF and produced EBs. Then, these bodies were differentiated to endoderm and then hepatocyte-like cells on 3D scaffold with and without miR-210 enforced expression.

We found that overexpressing miR-210 increased the expression of FOXA2 and SOX17 compared to the untransduced control group. Thus, these expression patterns confirmed the alteration of hiPSCs transduced by miR210 to endodermal cell lines. These results are consistent with several previous studies [5, 32–34]. Oct-4 expression, indicative of pluripotency, decreased in our endoderm cells, which was not evaluated in the study by Song et al. [5]. Additionally, in the three-step differentiation protocol explained above, miR-210 overexpression led to a significant hepatocyte differentiation based on analyzing hepatocyte markers such as SGPT and urea after 9 and 18 days. Also, SGOT levels revealed a significant difference only on day 18. AFP expression level reached the peak on day 9, indicating hepatoblast fate commitments of HLCs, and as the maturation proceeded, its level fell on day 18 according to hepG2. These factors used together in our 3D-miR group led to the formation of cells, resembling hepG2 cells as control positive cells.

Immunocytochemistry staining showed a mild increase in ALB and LDL-uptake ratio in 3D-miR compared to the 3D-non miR group. Besides, glycogen storage displayed significant effects of miR-210 on hepatocyte differentiation. However, there were slightly lower levels of AFP in the 3D-miR group, which was not reliable, and there were no significant differences in lipid content between those two groups. Yu et al. reported hepatocyte differentiation of hiPSCs during their differentiation cell density decreased while the cytoplasmic: nuclear ratio increased, and they became more flattened after 23 days. However, in our study, there were no significant morphological differences between 3D-miR and control groups after 18 days, except for the 3D-miR group, which changed to less flattened cells with the spherical process under SEM. Albumin immunocytochemistry, glycogen synthesis staining outcomes were in line with the current study but unlikely AFP continued to increase until 23 days of differentiation. HLCs compared to human hepatocytes expressed higher levels of AFP but lower levels of albumin mRNA. Conversely, urea production was not comparable with their positive control group [34]. In another experiment, Chinese researchers reported AFP and albumin expression after 7 days of hiPSC differentiation in a stepwise protocol [5].

Some studies measured SGPT and SGOT levels to assess hepatocyte function. Kazemnejad et al. reported an increase in the SGPT and SGOT concentrations 21 days after human bone marrow mesenchymal stem cell differentiation to HLCs [35]. However, we also observed a significant elevation in the 3D-miR group for SGOT and SGPT, compared to the 3D-non miR group, which showed no significant differences with hePG2 lines. However, in the 3D-non miR group, there was a reduction in SGPT on the 18th compared to the 9th day. This discrepancy could be related to different stem cells and scaffold structures. Consistent with our results, Asgari et al. induced hepatocyte differentiation of iPSC by approximately the same protocol used in our study with some differences in lacking a feeder layer in the culture medium and using PLC as positive control cells [36]. Our HLCs manifested the same function and expression pattern as previous studies utilizing other miRNAs and growth factors [14].

Several prior studies have evaluated the efficacy of different miRNAs on hepatic differentiation, such as miRNA-122 and miRNA-375, at various maturation levels, coming up with their potency [14, 24, 37–39]. When it comes to hypoxia, HIF is one of the main mediators, comprising two alpha and beta subunits. HIF*α* targets a proximal promoter region of miR-210, called hypoxia-responsive element [14], and positively regulates the miR-210 expression [40]. Subsequently, miR-210 interferes with the expression of some cellular hypoxia response genes. We focused on miR-210 as a master of hypoxic response and the remarkable specificity of the hypoxic signaling in miR-210 regulation. Hypoxia upregulates this miRNA in all cells and tissues [17]. Comparably, Liu et al. showed that miRNA-210 treatment before hADMSC transplantation could increase aminotransferase (AST, ALT) concentration in response to GAL/LPS challenge. Thus, the present study suggested an intensifying effect of miR-210 on stem cell efficacy [41]. However, hypoxia can affect both apoptosis and cell survival, leading miR-210 to act differently depending on the cellular context, the extent, and hypoxia duration [42].

Besides hypoxic-related features, previous studies established a close connection between miR-210 and ROS generation [43]. Of note, the exact mechanism by which miR-210 under hypoxia induces ROS production in different cell pathways is unclear [42, 44]. We observed oxidative stress altering among differentiation in 3D-miR and 3D-non miR groups under hypoxia niches, showing reduced ROS production in the 3D-miR group compared to the non-miR group. In addition, ROS production was markedly higher in the hepG2 cells compared to our miR groups. A high level of miR-210 might have a regressive role in ROS generation and a mediator role in antioxidant pathways, which results in cell survival under a hypoxic microenvironment, in line with many studies [41, 45, 46]. On the other hand, multiple investigations declared inverse results in various conditions based on the function of miR-210 on that cellular pathway. For instance, a positive feed-forward loop between ROS generation and miR-210 upregulation was reported in colorectal cancer cells and adipose-derived stem cells (ASCs) [20, 29, 47]. To interpret these differences, the downregulation of iron-sulfur cluster scaffold homolog and protein tyrosine phosphatase 1B should be noted in the cancer cells and not in the hiPSC-derived HLCs. We also applied a nanofiber scaffold, simulating the natural extra matrix environment of the cells. In line with our findings, human umbilical vein endothelial cells (HUVECs), cardiomyocytes, mesenchymal stem cells, and ischemic hind limbs were indicated to decrease ROS production [18, 20, 29].

Our study has some limitations. First, we could not identify the potential differentiation of PLLA/PCL distinctly due to being time and cost-consuming; thus, further studies can reach the most effective strategies and evaluate other structure to obtain perfect and efficient liver cells for in vivo regenerative medicine. Second, we did not show the viability and apoptosis of miR210 overexpressing cells due to nonsignificant data. Moreover, we did not evaluate ROS content using more specific methods such as DHF or red amplex, though it would be better to use these methods to evaluate ROS as the main finding in future studies. Overall, we first applied hypoxamiR as a lentiviral vector to attain hepatocyte differentiation on PLLA/PCL hybrid scaffold.

## 5. Conclusions

Transduction with a retrovirus containing miR-210 under oxidative stress conditions led to the differentiation of hiPSC based on the analysis of definitive endoderm gene marker expression and hepatocyte-specific marker production. As we previously confirmed the efficacy of the PLLA/PCL scaffold, this new method was examined on this 3D nano culture for the first time. Hence, we developed a novel and cost-effective approach to producing differentiated hepatocytes from hiPSCs. Future research must examine the risk of carcinogenesis regarding virus transduction to address the concerns about this technique and obtain desirable hepatocytes for in vivo regenerative medicine.

## Figures and Tables

**Table 1:** miRNA 1st-Strand cDNA synthesis.

miR-210	Stemloop	5′ GTC GTA TGG AGA GCA GGG TCC GAG GTA TTC GCA CTCCAT ACG ACC AGT GT 3′
	F	5′ TGATTAGCCCCTGCCCAC 3′
	R	5′ GAGCAGGGTCCGAGGT 3′
Snord 47 (internal control)	Stemloop	5′ GTCGTATGCAGAGCAGGGTCCGAGGTATTCGCACTGCATACGACAACCTC 3′
	F	5′ ATCACTGTAAAACCGTTCCA 3′
	R	5′ GAGCAGGGTCCGAGGT 3′

Amplicon length: 71 bp; F: forward; R: reverse.

**Table 2:** Primer sequences and conditions used for qRT-PCR of definitive endoderm gene markers.

Primer	Sequence	Annealing temperature (°C)
SOX17	F 5′-CAA GAT GCT GGG CAA GTC-3′	60
	R 5′-TGG TCC TGC ATG TGC TG-3′	
FOXA2	F 5′-AGC GAG TTA AAG TAT GCT GG-3′	58
	R 5′-GTA GCT GCT CCA GTC GGA-3′	
OCT-4	F 5′-TTCGCAAGCCCTCATTTCAC-3′	60
	R 5′-CCATCACCTCCACCACCTG-3′	
Beta2M	F 5′-ATG CCT GCC GTG TGA AC-3′	56
	R 5′ATC TTC AAA CCT CCA TGA TG-3	

SOX17: SRY-Box Transcription Factor 17; FOXA2: forkhead box protein A2; OCT-4: octamer-binding transcription factor 4; Beta2M; *β*2 microglobulin.

## Data Availability

Data can be obtained from the authors upon request.

## Abbreviations

hiPSCs: Human-induced pluripotent stem cells
PLLA/PCL: Poly-L-lactic acid/poly (*ε*-caprolactone)
IPS: Induced pluripotent stem cell
miR: MicroRNA
3D: Three-dimensional
SOX17: SRY-Box Transcription Factor 17
FOXA2: Forkhead box A2
OCT-4: Octamer-binding transcription factor 4
SEM: Scanning electron microscopy
ROS: Reactive oxygen species
AFP: Alpha-fetoprotein
LDL: Low-density lipoprotein
AST: Aspartate aminotransferase
ALT: Alanine aminotransferase
ALB: Albumin
DMEM: Dulbecco's modified Eagle's medium
FBS: Fetal bovine serum
PBS: Phosphate buffer saline
EB: Embryonic body
PAS: Periodic acid-Schiff
ICC: Immunocytochemistry
HLC: Hepatocytes-like cells.

## Supplementary Materials

Supplementary 1 Supplementary Figure S1: CDNA synthesis steps for miR-210.

Supplementary 2 Supplementary Table S1: mean of fold change (log) of IP, 3D, and 3D-miR and results of ANOVA, comparing the mean fold change (log) of IP, 3D, and 3D-miR in different groups (mean ± SD). Supplementary Table S2: concentration of AFP, AST activity, ALT secretion, and urea production in 3D-non miR and 3D-miR group on the 9th and 18th days; values are presented as mean ± SD. Supplementary Table S3: mean of ROS and results of ANOVA for comparison of the mean of ROS on days 9 and 18 in 3D-non miR and 3D-miR groups.
