# Supplementary figures and images for: Hypoxia-Induced miR-210 Overexpression Promotes the Differentiation of Human-Induced Pluripotent Stem Cells to Hepatocyte-Like Cells on Random Nanofiber Poly-L-Lactic Acid/Poly (ε-Caprolactone) Scaffolds
Source: Oxid Med Cell Longev. 2021 Nov 22;2021:4229721. doi: 10.1155/2021/4229721 (PMC8630456; doi:10.1155/2021/4229721)

Supplementary Figure S1

CDNA synthesis steps for miR-210

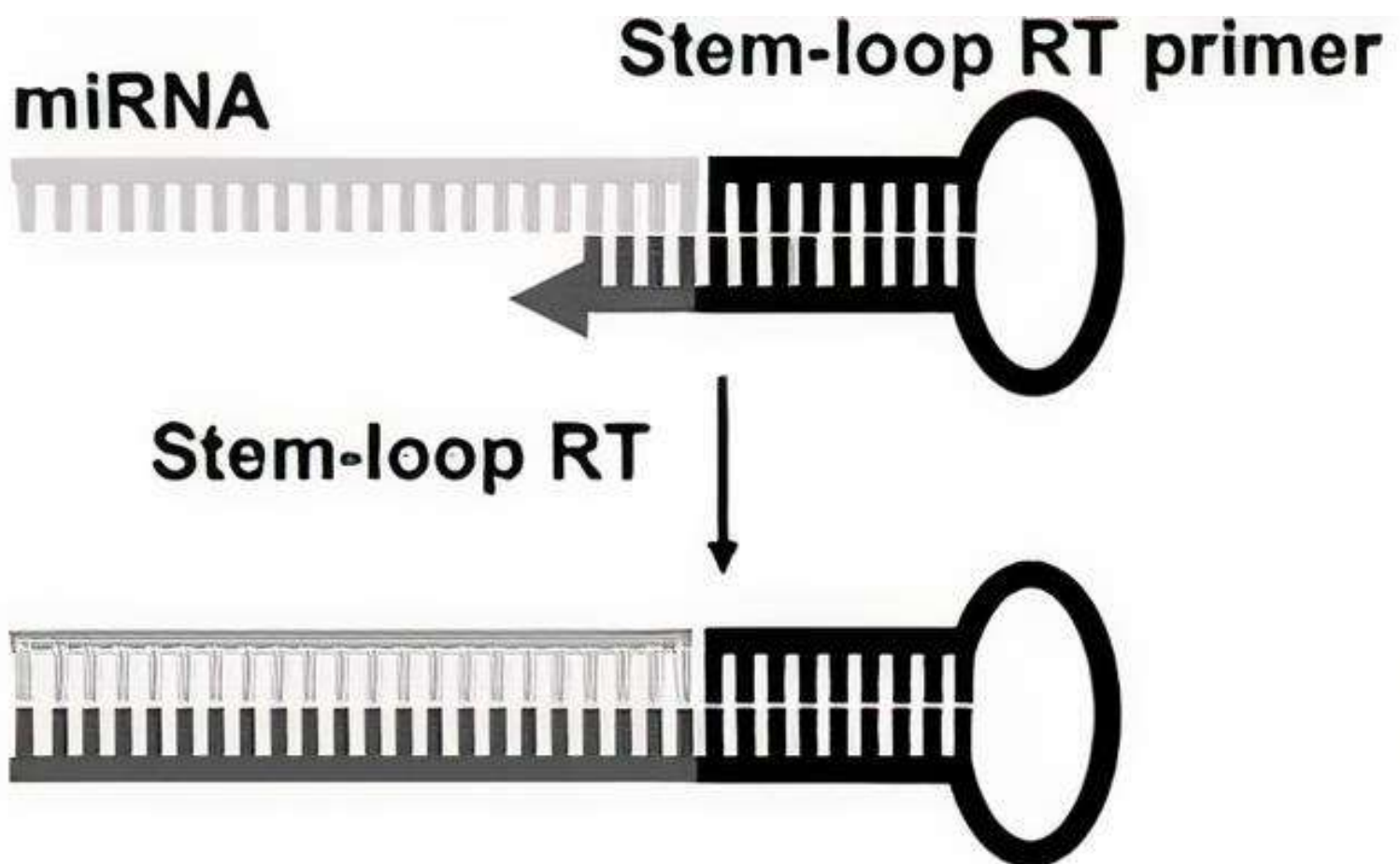

Supplement: Supplementary 1 — Supplementary Figure S1: CDNA synthesis steps for miR-210. [file 4229721.f1.pdf]
