# Supplementary material for: Hypoxia-Induced miR-210 Overexpression Promotes the Differentiation of Human-Induced Pluripotent Stem Cells to Hepatocyte-Like Cells on Random Nanofiber Poly-L-Lactic Acid/Poly (ε-Caprolactone) Scaffolds
Source: Oxid Med Cell Longev. 2021 Nov 22;2021:4229721. doi: 10.1155/2021/4229721 (PMC8630456; doi:10.1155/2021/4229721)
Supplement: Supplementary 2 — Supplementary Table S1: mean of fold change (log) of IP, 3D, and 3D-miR and results of ANOVA, comparing the mean fold change (log) of IP, 3D, and 3D-miR in different groups (mean ± SD). Supplementary Table S2: concentration of AFP, AST activity, ALT secretion, and urea production in 3D-non miR and 3D-miR group on the 9th and 18th days; values are presented as mean ± SD. Supplementary Table S3: mean of ROS and results of ANOVA for comparison of the mean of ROS on days 9 and 18 in 3D-non miR and 3D-miR groups. [file 4229721.f2.docx]

**Supplementary Table S1.** Mean of fold change (log) of IP, 3D, and 3D-miR and results of ANOVA for comparison of mean fold change (log) of IP, 3D, and 3D-miR in different groups (Mean ± SD)

| **Groups** | | **Mean ± SD** | ***p-value*** | **ANOVA comparison** | **p-value** |
| --- | --- | --- | --- | --- | --- |
| IPS | SOX 17 | -0.23 ± 0.03 | *<0.001* | SOX17>FOXA2 | 0.031 |
|  | FOX a2 | -0.29 ± 0.01 |  | SOX17<0CT-4 | <0.001 |
|  | 0CT-4 | 0.03 ± 0.01 |  | FOXA2<0CT-4 | <0.001 |
| 3D | SOX 17 | 0.50 ± 0.02 | *<0.001* | SOX17=FOXA2 | 0.122 |
|  | FOX a2 | 0.70 ± 0.01 |  | SOX17>0CT-4 | <0.001 |
|  | 0CT-4 | -1.33 ± 0.19 |  | FOXA2>0CT-4 | <0.001 |
| 3D-miR | SOX 17 | 0.94 ± 0.01 | *<0.001* | - | - |
|  | FOX a2 | 1.05 ± 0.01 |  |  |  |

Abbreviations: IPS, induced pluripotent stem cell; miR, microRNA; SOX17, SRY-Box Transcription Factor 17; FOXA2, Forkhead box protein A2; OCT-4; octamer-binding transcription factor 4; 3D, group culture on three-dimensional scaffold without miR-210 transduction; 3D-miR, group cultured on three-dimensional scaffold with miR-210 transduction

**Supplementary Table S2.** Concentration of AFP, AST activity, ALT secretion, and Urea production in 3D-non miR and 3D-miR group on 9th and 18th day, values are presented as Mean ± SD

| **AFP** | | | | | | | | | **Urea** | | | |
| --- | --- | --- | --- | --- | --- | --- | --- | --- | --- | --- | --- | --- |
| Day | | | | Mean ± SD | p-value | ANOVA comparison | | P- value | Mean ± SD | P-value | ANOVA comparison | P -value |
| 9 | | 3D-non miR | | 48.81 ± 0.61 | <0.001 | - | | - | 4.04 ± 0.12 | <0.001 | - | - |
|  |  | 3D-miR | | 84.12 ± 1.02 |  |  |  |  | 8.01 ± 0.08 |  |  |  |
| 18 | | 3D-non miR | | 32.33 ± 0.76 | <0.001 | 3D-non miR> 3D-miR | | <0.001 | 9.06 ± 0.17 | <0.001 | 3D-non miR< 3D-miR | <0.001 |
|  |  | 3D-miR | | 24.12 ± 1.02 |  | 3D-non miR>hepG2 | | <0.001 | 15.63 ± 0.40 |  | 3D-non miR<hepG2 | <0.001 |
|  |  | hepG2 | | 24.83 ± 1.26 |  | 3D-miR=hepG2 | | 0.694 | 16.05 ± 0.21 |  | 3D-miR=hepG2 | 0.230 |
| **AST** | | | | | | | |  | **ALT** | | | |
| Day | | | Mean ± SD | | P -value | | ANOVA comparison | P- value | Mean ± SD | P value | ANOVA comparison | P-value |
| 9 | 3D-non miR | | 5.03 ± 0.10 | | 0.998 | | - | - | 2.02 ± 0.12 | <0.001 | - | - |
|  | 3D-miR | | 5.03 ± 0.11 | |  |  |  |  | 3.03 ± 0.08 |  |  |  |
| 18 | 3D-non miR | | 13.76 ± 0.52 | | <0.001 | | 3D-non miR< 3D-miR | <0.001 | 1.03 ± 0.09 | <0.001 | 3D-non miR< 3D-miR | <0.001 |
|  | 3D-miR | | 18.02 ± 0.09 | |  |  | 3D-non miR<hepG2 | <0.001 | 3.03 ± 0.1 |  | 3D-non miR<hepG2 | <0.001 |
|  | hepG2 | | 18.97 ± 1.01 | |  |  | 3D-miR=hepG2 | 0.258 | 3.07 ± 0.15 |  | 3D-miR=hepG2 | 0.937 |

Abbreviations: AFP, Alpha-fetoprotein; AST, Aspartate aminotransferase; ALT, Alanine aminotransferase; 3D-non miR, group culture on three-dimensional scaffold without miR-210 transduction; 3D-miR, group cultured on three-dimensional scaffold with miR-210 transduction; hepG2, used as positive control group

**Supplementary Table S3.** Mean of ROS and results of ANOVA for comparison of the mean of ROS on days 9 and 18 in 3D-non miR and 3D-miR groups

| groups | | Mean ± SD | p-value | ANOVA comparison | p-value |
| --- | --- | --- | --- | --- | --- |
| 9 | 3D- miR | 3.63±0.09 | <0.001 | - | - |
|  | 3D- non miR | 8.74±0.05 |  |  |  |
| 18 | 3D miR | 2.89±0.06 | <0.001 | 3D –miR< 3D-non miR | <0.001 |
|  | 3D-non miR | 6.64±0.11 |  | 3D –miR< hepG2 | <0.001 |
|  | hepG2 | 6.59±0.11 |  | 3D-non miR= hepG2 | 0.820 |

Abbreviations: ROS, Reactive oxygen species; 3D-non miR, group culture on three-dimensional scaffold without miR-210 transduction; 3D-miR, group cultured on three-dimensional scaffold with miR-210 transduction
